# Supplementary material for: A lentiviral vector for the production of T cells with an inducible transgene and a constitutively expressed tumour-targeting receptor
Source: Nat Biomed Eng. 2023 Apr 17;7(9):1063–80. doi: 10.1038/s41551-023-01013-5 (PMC10504085; doi:10.1038/s41551-023-01013-5)
Supplement: Supplementary file 1 — Supplementary Table 1. [file 41551_2023_1013_MOESM1_ESM.pdf]

nature biomedical engineering

Article https://doi.org/10.1038/s41551-023-01013-5

A lentiviral vector for the production of T cells with an inducible transgene and a constitutively expressed tumour-targeting receptor

In the format provided by the authors and unedited

Supplemetary Table 1 | Sequences of components encoded in lentiviral vectors.

**Vector Component**	**Sequence**
$(NFAT)_6$ response elements-IL-2 minimal promoter (abbreviated 6xNFAT)	GGAGGAAAAACTGTTTCATACAGAAGGCGTGGAGGAAAAACTG<br>TTTCATACAGAAGGCGTGGAGGAAAAACTGTTTCATACAGAAG<br>GCGTGGAGGAAAAACTGTTTCATACAGAAGGCGTGGAGGAAAA<br>ACTGTTTCATACAGAAGGCGTGGAGGAAAAACTGTTTCATACA<br>GAAGGCGTGACATTTTGACACCCCCATAATATTTTTCCAGAATT<br>AACAGTATAAATTGCATCTCTTGTTCAAGAGTTCCCTATCACTC<br>TCTTTAATCACTACTCACAGTAACCTCAACTCCTGC
BGH Polyadenylation sequence	CTGTGCCTTCTAGTTGCCAGCCATCTGTTGTTTGCCCCTCCCC<br>CGTGCCTTCCTTGACCCTGGAAGGTGCCACTCCCACTGTCCTT<br>TCCTAATAAAATGAGGAAATTGCATCGCATTGTCTGAGTAGGTG<br>TCATTCTATTCTGGGGGGTGGGGTGGGGCAGGACAGCAAGGG<br>GGAGGATTGGGAAGACAATAGCAGGCATGCTGGGGATGCGGT<br>GGGCTCTATGG
SPA + human transcr. pausing site	CTAGCAATAAAAGATCTTTATTTTCATTAGATCTGTGTGTTGGTT<br>TTTTGTGTGTCTAGTTAATTAAGGTGGGGCAGTGGGGGCCAAC<br>TTGTCCTTACCCAGAGTGCAGGTGTGTGGAGATCCCTCCTGCC<br>TTGACATTGAGCAGCCTTAGAGGGTGGGGGAGGCTCAGGGGT<br>CAGGTCTCTGTTCCTGCTTATTGGGGAGTTCCTGGCCTGGCCC<br>TTCTATGTCTCCCCAGGTACCCCAGTTTTTCTGGGTTCACCCAG<br>AGTGCAGATGCTTGAGGAGGTGGGAAGGGACTATTTGGGGGT<br>GTCTGGCTCAGGTGCCATGCCTCACTGGGGCTGGTTGGCACC<br>TGCATTTCCTGGGAGTGGGGCTGTCTCAGGGTAGCTGGGCAC<br>GGTGTTCCCTTGAGTGGGGGTGTAGTGGGTGTTCCTAGCTGC<br>CACGCCTTTGCCTTCACCTATGGGATCGTGGCTGTCAG
TAX Protein	ATGGCACACTTTCCTGGGTTTGGGCAGTCACTGCTCTTTGGGT<br>ACCCCGTTTATGTATTCGGCGACTGTGTTCAAGGAGACTGGTG<br>TCCTATCTCAGGCGGTCTCTGTTCCGCTCGACTCCACCGGCAT<br>GCACTCCTCGCGACATGTCCCGAGCATCAGATAACCTGGGATC<br>CAATCGACGGAAGGGTGATAGGCTCTGCCCTGCAGTTTCTCAT<br>CCCTCGCCTGCCCAGCTTTCCCACCCAGCGAACCTCCAAAACC<br>TTGAAGGTCTTGACCCCTCCAATCACACATACTACCCCTAACAT<br>CCCGCCTTCCTTCCTGCAAGCCATGCGAAAATACAGCCCCTTC<br>AGAAACGGTTACATGGAACCTACTTTGGGTCAGCACCTGCCTA<br>CATTGTCCTTTCCGGACCCCGGCCTGCGACCGCAGAACCTGTA<br>TACACTGTGGGGCGGCTCTGTCGTCTGTATGTACCTCTACCAG<br>CTGTCACCGCCCATCACGTGGCCCCTGTTGCCCCATGTAATAT<br>TTTGCCACCCAGGACAGCTCGGCGCCTTTCTGACAAACGTCCC<br>TTATAAACGAATCGAAAAACTCCTGTATAAAATTTCACTGACCA<br>CCGGAGCCCTGATAATACTGCCTGAAGACTGCCTGCCAACCAC<br>TCTGTTTCAACCCGCTAGGGCTCCCGTCACCCTCACTGCTTGG<br>CAGAACGGACTCCTGCCCTTCCATTCCACCCTCACAACTCCAG<br>GGTTGATTTGGACATTTACCGATGGAACTCCTATGATTTCCGGA<br>CCTTGTCCCAAGGACGGACAGCCATCCTTGGTGCTCCAATCCT

	CCTCCTTTATTTTCCACAAGTTTCAAACGAAGGCATACCATCCT AGCTTCCTGCTGAGCCATGGACTGATACAGTACTCTTCTTTTCA TAATCTGCACCTGTTGTTTGAGGAGTATACTAATATCCCAATCA GCCTCCTCTTTAATGAAAAAGAGGCGGACGACAATGATCATGA GCCGCAGATCTCACCTGGCGGGCTGGAGCCCCTTAGCGAAAA GCATTTTCGCGAAACAGAGGTC
U6	GAGGGCCTATTTCCCATGATTCCTTCATATTTGCATATACGATA CAAGGCTGTTAGAGAGATAATTGGAATTAATTTGACTGTAAACA CAAAGATATTAGTACAAAATACGTGACGTAGAAAGTAATAATTT CTTGGGTAGTTTGCAGTTTTAAAATTATGTTTTAAAATGGACTAT CATATGCTTACCGTAACTTGAAAGTATTTCGATTTCTTGGCTTTA TATATCTTGTGGAAAGGAC
SFFV	GTAACGCCATTTTGCAAGGCATGGAAAAATACCAAACCAAGAA TAGAGAAGTTCAGATCAAGGGCGGGTACATGAAAATAGCTAAC GTTGGGCCAAACAGGATATCTGCGGTGAGCAGTTTCGGCCCC GGCCCGGGGCCAAGAACAGATGGTCACCGCAGTTTCGGCCCC GGCCCGAGGCCAAGAACAGATGGTCCCCAGATATGGCCCAAC CCTCAGCAGTTTCTTAAGACCCATCAGATGTTTCCAGGCTCCC CCAAGGACCTGAAATGACCCTGCGCCTTATTTGAATTAACCAAT CAGCCTGCTTCTCGCTTCTGTTCGCGCGCTTCTGCTTCCCGAG CTCTATAAAAGAGCTCACAACCCCTCACTCGGCGCGCCAGTCC TCCGACAGACTGAGTCGGCCGG
NovB2	ATGACCAACATGAGCTGCGCCTACGAGCTGATCAAGAGCCTGC CCGCCAAGCTGGAACAGCTGGCCCAGGAAACCCAGGCCACCA TCCAGACCCTGATGATCGCCGACCCCAACGTGAACAAGGACCT GCGGGCCTTCTGCGAGTTCCTGACCGTGCAGCACCAGCGGGC CTACAGAGCCACCAACAGCCTGCTGATTAAGCCCAGAGTGGC CGCTGCCCTGAGAGGCGAGGAACTGGATCTGGGAGAGGCCG ATGTGGCCGCTAGAGTGCGGCAGCTGAAACAGCAGCTGGCCG AGCTGGAAATGGAAATCAAGCCCGGCCATCAGCAGGTGGCCC AGGTGTCCGGAAGAAGAAAAGCCGCCGCTGCCGCTCCTGTGG CTCAGCTGGGAAGAGTGGGAGTCGTGAACGAG
CMV-5’LTR promoter	GTGATGCGGTTTTGGCAGTACATCAATGGGCGTGGATAGCGGT TTGACTCACGGGGATTTCCAAGTCTCCACCCCATTGACGTCAA TGGGAGTTTGTTTTGGCACCAAAATCAACGGGACTTTCCAAAAT GTCGTAACAACTCCGCCCCATTGACGCAAATGGGCGGTAGGC GTGTACGGTGGGAGGTCTATATAAGCAGAGCT
