# Supplementary material for: A lentiviral vector for the production of T cells with an inducible transgene and a constitutively expressed tumour-targeting receptor
Source: Nat Biomed Eng. 2023 Apr 17;7(9):1063–80. doi: 10.1038/s41551-023-01013-5 (PMC10504085; doi:10.1038/s41551-023-01013-5)
Supplement: Supplementary file 2 — Reporting Summary [file 41551_2023_1013_MOESM2_ESM.pdf]

nature portfolio

Corresponding author(s): Melita Irving and George Coukos

Last updated by author(s): Feb 20, 2023

Reporting Summary

Nature Portfolio wishes to improve the reproducibility of the work that we publish. This form provides structure for consistency and transparency in reporting. For further information on Nature Portfolio policies, see our Editorial Policies and the Editorial Policy Checklist.

Statistics

For all statistical analyses, confirm that the following items are present in the figure legend, table legend, main text, or Methods section.

n/a	Confirmed	
☐	☒	The exact sample size (*n*) for each experimental group/condition, given as a discrete number and unit of measurement
☐	☒	A statement on whether measurements were taken from distinct samples or whether the same sample was measured repeatedly
☐	☒	The statistical test(s) used AND whether they are one- or two-sided<br>*Only common tests should be described solely by name; describe more complex techniques in the Methods section.*
☒	☐	A description of all covariates tested
☐	☒	A description of any assumptions or corrections, such as tests of normality and adjustment for multiple comparisons
☐	☒	A full description of the statistical parameters including central tendency (e.g. means) or other basic estimates (e.g. regression coefficient) AND variation (e.g. standard deviation) or associated estimates of uncertainty (e.g. confidence intervals)
☐	☒	For null hypothesis testing, the test statistic (e.g. *F*, *t*, *r*) with confidence intervals, effect sizes, degrees of freedom and *P* value noted<br>*Give P values as exact values whenever suitable.*
☒	☐	For Bayesian analysis, information on the choice of priors and Markov chain Monte Carlo settings
☒	☐	For hierarchical and complex designs, identification of the appropriate level for tests and full reporting of outcomes
☒	☐	Estimates of effect sizes (e.g. Cohen's *d*, Pearson's *r*), indicating how they were calculated

Our web collection on statistics for biologists contains articles on many of the points above.

Software and code

Policy information about availability of computer code

Data collection	Incucyte Instrument, BD LSR II FACS, BD LSR SORP FACS, In-Vivo Xtreme system (Bruker Corp.), Western Blot Imager (Fusion, Vilber Lourmat). HIDEX.
Data analysis	IncuCyte Zoom 2016A Data analysis (Essen Bioscience), FACS DIVA Software, Microsoft Excel 2019, GraphPad Prism v8, FlowJo X, Molecular Imaging (MI) software (MI, Bruker Corp.), ImageJ software (pixel intensity of the bands). Hidex software for bioluminescence.

For manuscripts utilizing custom algorithms or software that are central to the research but not yet described in published literature, software must be made available to editors and reviewers. We strongly encourage code deposition in a community repository (e.g. GitHub). See the Nature Portfolio guidelines for submitting code & software for further information.

Data

Policy information about availability of data

All manuscripts must include a data availability statement. This statement should provide the following information, where applicable:

- Accession codes, unique identifiers, or web links for publicly available datasets
- A description of any restrictions on data availability
- For clinical datasets or third party data, please ensure that the statement adheres to our policy

The main data supporting the findings of this study are available within the article and its Supplementary Information. Source data for the figures are provided with this paper. All raw data generated during the study are available from the corresponding authors on request.

Field-specific reporting

Please select the one below that is the best fit for your research. If you are not sure, read the appropriate sections before making your selection.

☒ Life sciences ☐ Behavioural & social sciences ☐ Ecological, evolutionary & environmental sciences

For a reference copy of the document with all sections, see nature.com/documents/nr-reporting-summary-flat.pdf

Life sciences study design

All studies must disclose on these points even when the disclosure is negative.

Sample size	For the in vitro studies, the number of healthy donors and/or technical replicates were chosen according to the complexity of the assay and for the expected biological variability. For the in vivo studies, a maximum of 5 million CAR T cells per mouse were needed. We achieved a sample size of 5 animal per treatment group, which proved to be sufficient to reproducibly observe statistically significant differences.
Data exclusions	For the in vivo studies, upon calipering, outlier mice with extreme burdens (either too high or too low compared to the average) were excluded from the experiment before CAR-T-cell transfer. No mice were excluded afterwards at any point.
Replication	All attempts at replication were successful.
Randomization	Tumor burden was evaluated by calipering the same day of CAR T cell transfer. Outlier mice with extreme burdens (either too high or too low compared to the average) were excluded from the experiment before CAR T cell transfer. No mice were excluded afterwards at any point. Following tumor burden measure, mice were assigned into treatment groups such that each group had the same overall average tumor volume. Buffy coats and apheresis filters were obtained from anonymous donors.
Blinding	An independent investigator verified caliper measurements in a blinded fashion. The analysis of data (the plotting of pre-recorded tumour volumes at end of study) was performed in a non-blinded manner.

Reporting for specific materials, systems and methods

We require information from authors about some types of materials, experimental systems and methods used in many studies. Here, indicate whether each material, system or method listed is relevant to your study. If you are not sure if a list item applies to your research, read the appropriate section before selecting a response.

Materials & experimental systems

n/a	Involved in the study	
☐	☒	Antibodies
☐	☒	Eukaryotic cell lines
☒	☐	Palaeontology and archaeology
☐	☒	Animals and other organisms
☐	☒	Human research participants
☒	☐	Clinical data
☒	☐	Dual use research of concern

Methods

n/a	Involved in the study	
☒	☐	ChIP-seq
☐	☒	Flow cytometry
☒	☐	MRI-based neuroimaging

Antibodies

Antibodies used	Antibodies were titrated for optimal staining. Aqua live Dye BV510 (cat L34966, lot no 1899019, Invitrogen) and near-IR fluorescent reactive dye (APC Cy-7) (cat L34976A, Invitrogen, lot no 2379385) were used to assess viability. AlexaFluo 647-conjugated anti-mouse F(ab)' (cat 115-606-072, lot no 143040, Jackson Immuno Research) was used fror CAR detection. PE-labeled anti-human PSMA (clone LNI-17, cat 342503, lot no B211499, Biolegend) and PE Isotype mouse-anti-IgG1, k chain (clone MOPC-21, cat 400114, lot no B307873, Biolegend) were used to evaluated PSMA expression on cell line. PE-labeled HLA-A2/NY-ESO-1(157-165) tetramer (batch number 011220, PTCF at UNIL) was used to evaluate TCR expression. PE-labeld anti human NGFR (clone ME20.4, cat 345105, lot no B262596, Biolegend) was used to evaluate transduction efficiency. Anti-human HPK1 (rabbit monoclonal clone EP6430Y, cat ab33910, Abcam) was used to detect MAP4K1/HPK1 levels with western blot analysis. Anti-human-beta Actin (cat sc-47778, Santa Cruz) was used to detect beta-Actin as control for wester blot. PE-labled anti-human PAN TCR (cat B49177, lot no 200029, Beckman Coulter) was used to detect TCR expression on human T cells.
Validation	Antibody-concentration validation was empirically determined in the lab.

Eukaryotic cell lines

Policy information about cell lines

Cell line source(s)	293T, Jurkat, Saos, A375, Me275, Na8 and Bajb from ATCC. PC3-PIP from Dr. Rosato, University of Padova. Jurkat 6x-NFAT mCherry were engineered in the lab. PC3 and PC3-PIP CD19+ were engineered in the lab, provided by Prof. Jannick Muller.
Authentication	COA provided with cell line by ATCC. Properties pertinent to experiment (such as PSMA expression) were confirmed by flow cytometry.
Mycoplasma contamination	All cell lines were routinely tested for mycoplasma contamination, and found to be negative.
Commonly misidentified lines (See ICLAC register)	No commonly misidentified cell lines were used.

Animals and other organisms

Policy information about studies involving animals; ARRIVE guidelines recommended for reporting animal research

Laboratory animals	NSG male mice, 8–12 weeks old, were bred and housed in a SOPF animal facility.
Wild animals	The study did not involve wild animals.
Field-collected samples	The study did not involve samples collected from the field.
Ethics oversight	All in vivo experiments were conducted in accordance with and approval from the Service of Consumer and Veterinary Affairs (SCAV) of the Canton of Vaud (Switzerland), license VD3414.

Note that full information on the approval of the study protocol must also be provided in the manuscript.

Human research participants

Policy information about studies involving human research participants

Population characteristics	Buffy coats and apheresis filters from anonymous healthy donors were collected with informed consent of the donors, and genetically engineered with Ethics Approval from the Canton of Vaud.
Recruitment	N/A
Ethics oversight	Ethics approval from the Canton of Vaud to the laboratory of Prof. George Coukos allowed gene engineering of primary human T cells.

Note that full information on the approval of the study protocol must also be provided in the manuscript.

Flow Cytometry

Plots

Confirm that:

☒ The axis labels state the marker and fluorochrome used (e.g. CD4-FITC).

☒ The axis scales are clearly visible. Include numbers along axes only for bottom left plot of group (a 'group' is an analysis of identical markers).

☒ All plots are contour plots with outliers or pseudocolor plots.

☒ A numerical value for number of cells or percentage (with statistics) is provided.

Methodology

Sample preparation	Primary human T cells were isolated from the peripheral blood mononuclear cells (PBMCs) of healthy donors (HDs; prepared as buffycoats or apheresis filters). All blood samples were collected with informed consent of the HDs, and genetically-engineered with Ethics Approval from the Canton of Vaud to the laboratory of Prof. George Coukos. Total PBMCs were obtained via Lymphoprep (Axonlab) separation solution, using a standard protocol of centrifugation. CD4+ and CD8+ T cells were isolated using a magnetic bead-based negative selection kit following the manufacturer's recommendations (easySEP, Stem Cell technology). Purified CD4+ and CD8+ T cells were cultured at a 1:1 ratio in RPMI-1640 with Glutamax, supplemented with 10% heat-inactivated FBS, 100 U/mL penicillin, 100 µg/mL streptomycin sulfate, and stimulated with anti-CD3 and anti-CD28 monoclonal antibody (mAb)-coated-beads (Lifetechnologies) in a ratio of 1:2, T cells: beads. For staining preparation, cells were washed once and resuspended in FACS buffer containing LIVE/DEAD dye and the antibody cocktail. Cells were incubated at 4 degrees for 30 minutes and washed twice before acquisition. Cells were not fixed prior to acquisition.
Instrument	LSR II, BD

Software	Collection: FACS DIVA<br>Analysis: FlowJo X
Cell population abundance	T-cell purification from PBMCs by magnetic beads was validated by flow for CD4+/CD8+ cells. T-cell purity was >99%.
Gating strategy	The starting cell population was gated on a linear SSC-A/FSC-A plot. Single cells were discriminated on a linear FSC-H/FSC-A plot. Live cells were determined by exclusion from positive live/dead-stained cells. Positive/negative populations were determined with negative controls.

☐ Tick this box to confirm that a figure exemplifying the gating strategy is provided in the Supplementary Information.
