# Supplementary figures and images for: A lentiviral vector for the production of T cells with an inducible transgene and a constitutively expressed tumour-targeting receptor
Source: Nat Biomed Eng. 2023 Apr 17;7(9):1063–80. doi: 10.1038/s41551-023-01013-5 (PMC10504085; doi:10.1038/s41551-023-01013-5)

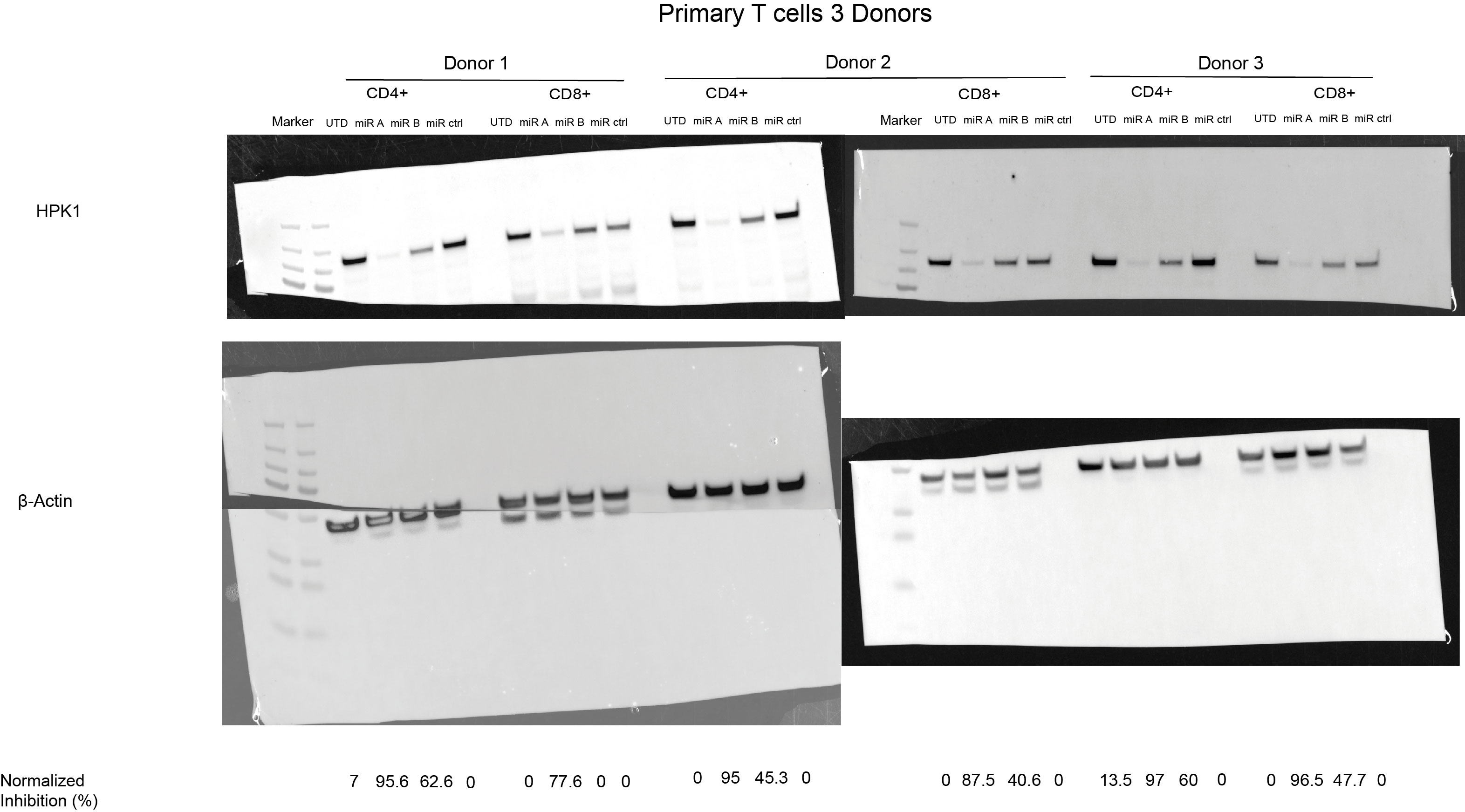

Supplement: SD for Fig. 6 — Unprocessed western blot. [file 41551_2023_1013_MOESM5_ESM.jpg]

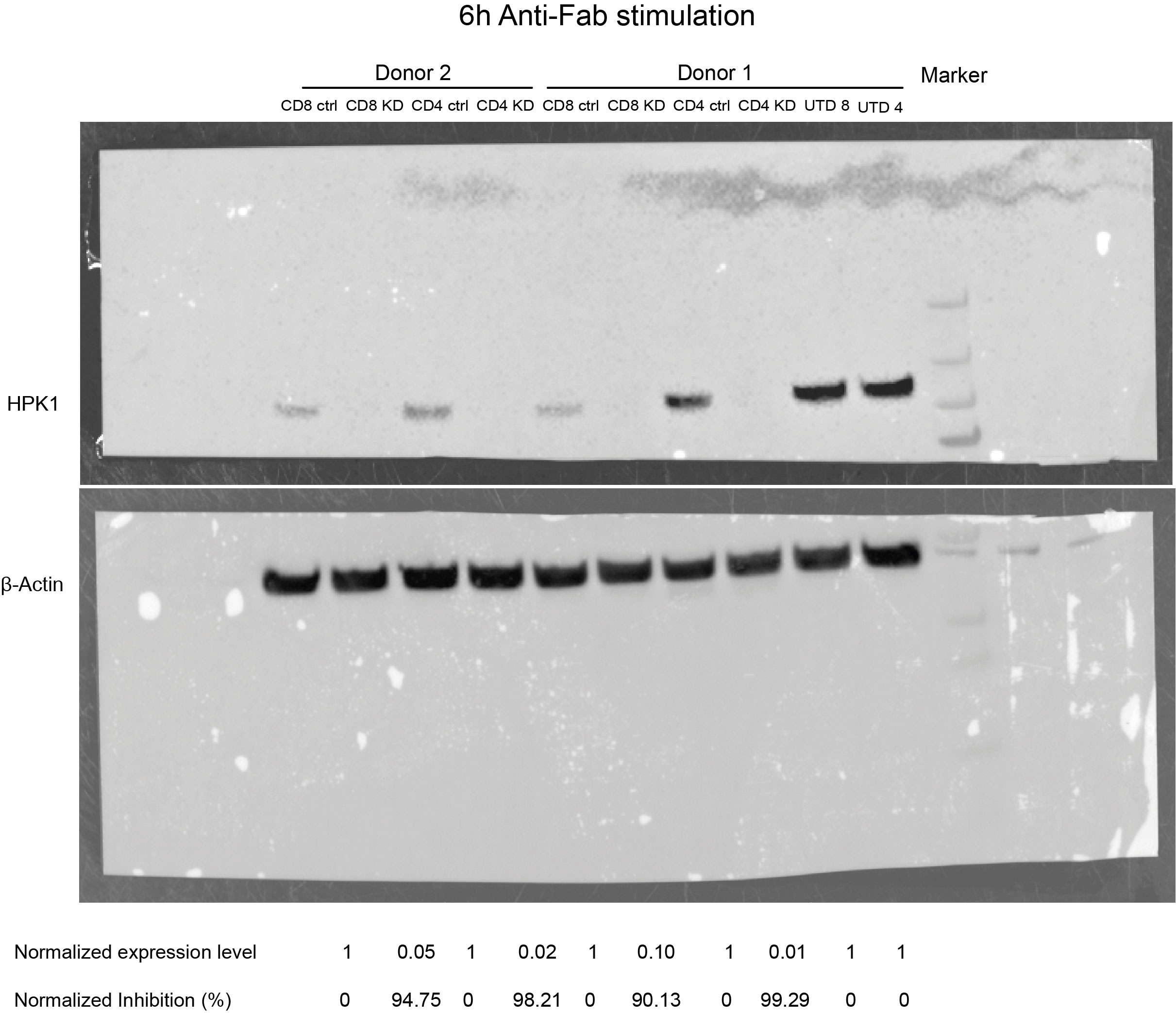

Supplement: SD for Fig. 7 — Unprocessed western blot. [file 41551_2023_1013_MOESM6_ESM.jpg]
